# Echocardiography Does Not Delay Surgery in Elderly Patients with Hip Fractures, and Pulmonary Hypertension and Decreased Left Ventricular Ejection Fraction Are Associated with In-Hospital Mortality

**DOI:** 10.3390/jcm15114284

**Published:** 2026-06-01

**Authors:** Carlo Rostagno, Alessandro Cartei, Gaia Rubbieri, Alice Ceccofiglio, Giulio Maria Mannarino, Roberto Civinini

**Affiliations:** 1 Dipartimento Medicina Sperimentale e Clinica, Università di Firenze, 50134 Florence, Italy; 2 SOD Geriatria, AOU Careggi, 50134 Florence, Italy; carteia@aou-careggi.toscana.it (A.C.); rubbierig@aou-careggi.toscana.it (G.R.); ceccofiglioa@aou-careggi.it (A.C.); mannarinog@aou-careggi.toscana.it (G.M.M.); 3 SOD Ortopedia e Traumatologia, AOU Careggi, 50134 Florence, Italy; roberto.civinini@unifi.it

**Keywords:** hip fracture, echocardiography, outcome

## Abstract

**Background:** Cardiovascular complications are the main cause of early mortality in elderly patients after hip fracture surgery. Echocardiography, although suggested by guidelines to improve risk stratification, is frequently omitted due to the risk of delaying surgery. The aim of the study was to evaluate whether in a multidisciplinary hip fracture unit, echocardiography can be performed without a delay in surgery. The secondary endpoint was to research the possible association between echocardiographic abnormalities and in-hospital mortality. **Methods:** The study included hip fracture patients aged > 70 admitted in the period 1 January 2019 to 31 December 2024, to the hip fracture unit of a tertiary teaching hospital. Echocardiography was indicated according to clinical criteria (detection of heart murmur, pathological electrocardiographic changes, known heart disease and the presence of ≥3 coronary risk factors). In the study, there were 2272 patients; 1593 had indications for preoperative echocardiography, which was performed in 1502. Mean age was significantly higher in the ECHO group than in the NO ECHO group (85.4 ± 8 vs. 80.5 ± 11 years, *p* < 0.0001). ECHO group patients more frequently had at least two comorbidities and more severe functional impairment. In-hospital mortality was 7.3% in ECHO patients compared to 2.3% in NO ECHO patients. **Results:** Multivariate analysis showed that decreased left ventricular ejection fraction and pulmonary hypertension, as well as age, anemia, and reduced functional capacity expressed as lost BADL, were associated with in-hospital mortality. **Conclusions:** Echocardiography identifies a population at a high risk of in-hospital mortality, three times higher compared to the group of NO ECHO patients. In those who underwent echocardiography, a reduced left ventricular ejection fraction and an increase in pulmonary pressure were associated with in-hospital mortality.

## 1. Introduction

Routine preoperative echocardiography before non-cardiac surgery has not been demonstrated to improve outcomes in large cohort studies [1,2], although specific abnormalities such as degree of mitral regurgitation or aortic stenosis, LVEF and E/e’ ratio may be associated with increased mortality in patients undergoing intermediate- or high-risk non-cardiac surgery [3]. Cardiovascular complications, mainly heart failure and myocardial infarction/damage, are the leading causes of death and prolonged hospitalization after hip surgery [4]. Risk stratification according to the AHA/ACC or ESC guidelines in patients with known heart disease can be difficult since at least 35% of patients have cognitive impairment and/or moderate/severe functional limitations [5,6]. Therefore, symptoms related to heart failure or chronic coronary artery disease may be underestimated. Early surgery, within 24–48 h of trauma, is associated with more favorable clinical outcomes [7,8]. According to SIGN guidelines, echocardiography is recommended to better stratify surgical risk and define the anesthesiologist’s strategy in suspected aortic stenosis if examination does not delay surgery [9]. Substantial changes in perioperative management have been reported in no less than 20% of hip fracture patients after echocardiography. Moderate to severe valvular heart disease (mainly aortic stenosis or mitral regurgitation) is rarely found by chance at hospital admission or has not been re-evaluated for a long time [10,11]. Moreover, in patients with known heart failure or unexplained poor functional capacity, the assessment of left ventricular function and filling pressures may guide therapeutic optimization to limit the postoperative complication rate. Results of previous investigations about preoperative echocardiography in hip fracture are contrasting. The main concern is related to an unacceptable delay in treatment and a related increase in complications and mortality [12,13,14]. Canty et al. [15] compared high-risk cardiac patients who underwent preoperative transthoracic echocardiography (TTE) to a randomized cohort who had similar cardiac risk but did not undergo preoperative TTE. Mortality at 30 days (4.7% vs. 15.2%, *p* = 0.047) and 12 months after surgery (17.1% vs. 33.3%, *p* = 0.031) was lower in patients who underwent echocardiography. Among 354 patients who underwent TTE before hip surgery, age, history of CAD, presence of moderate–severe aortic stenosis and LVEF < 50% were independent predictors of mortality [16]. Higher average E/e’ values, lower left ventricular (LV) ejection fraction, and higher prevalence of significant mitral regurgitation were related to the risk of major cardiovascular events or death in 1453 patients (51% male; age, 67 ± 16) who underwent intermediate- or high-risk major abdominal surgery or orthopedic surgery [3]. A large retrospective study including more than 25,000 propensity score-matched patients did not show different in-hospital mortality between echocardiography-screened versus non-screened patients. A greater average time to surgery without significant advantages in in-hospital outcome were reported by AbuSharar et al. [17]. The study, however, included only 42 patients, 22% of whom had ACC/AHA indications for preoperative echocardiogram. The aim of the present study was to evaluate whether in a multidisciplinary hip fracture management setting, time to surgery was delayed by echocardiography, when needed. The secondary endpoint was the effects of examination on in-hospital mortality and length of stay. Finally, we investigated the relation between specific echocardiographic abnormalities and in-hospital mortality.

## 2. Materials and Methods

The study was a subproject of an investigation of the Italian Health Ministry and Regione Toscana—RF-2010-2316600. It was approved by the Ethical Committee of Area Vasta Centro e Regione Toscana (approval code: 13556_oss, date of approval: 11 September 2018). Written informed consent to treatment and collection of clinical data for research purposes was obtained at admission. The study was conducted according to STROBE statements and performed in line with the principles of the Declaration of Helsinki. 

The aim of the study was to evaluate whether in a multidisciplinary hip fracture unit, echocardiography can be performed without a delay in surgery in elderly patients hospitalized for a hip fragility fracture. Moreover, we evaluated the possible relation between echocardiographic abnormalities and in-hospital mortality. Finally, we compared the outcomes between patients who underwent preoperative echocardiography and those who were referred for surgery without echocardiographic examination.

### 2.1. Preoperative Evaluation

All patients with a hip fracture admitted to a tertiary teaching hospital between 1 January 2019 and 31 December 2024 were enrolled in the study. The patients were followed by a multidisciplinary group for fragility fractures. At admission each patient was carefully evaluated by the Internal Medicine–geriatric specialist (general clinical conditions, presence of comorbidities and ongoing medical treatment). For each patient, demographic data (age and sex) and the presence of comorbidities (arterial hypertension, atrial fibrillation, ischemic heart disease, heart failure, COPD, diabetes, both peripheral and cerebral vasculopathy, neoplasms, renal failure, anemia -Hb < 10 g/dL, osteoporosis, ≥2 diseases, and cognitive impairment) were recorded in the database. Finally, functional capacity prior to trauma was assessed using the Basic Activities of Daily Living Assessment Scale (BADL), which was easily recorded at the admission of patients. We were not able to perform objective evaluation of frailty with handgrip, and the clinically evaluated frailty score overlapped with included data.

Echocardiography was performed usually within 24 h from admission with an ESAOTE MY LAB 40 echocardiograph (ESAOTE, Genova, Italy). According to the protocol of the study, echocardiography was indicated in patients with newly detected or known systolic murmurs, ECG abnormalities, known heart disease or the presence of ≥3 coronary risk factors. Echocardiography was performed according to ASE/EACVI recommendations [18] with a standardized sequence of imaging. Recorded images were reviewed by a senior cardiologist. In patients with atrial fibrillation, we considered the mean of 5 measurements for each parameter.

Left ventricular ejection fraction was measured using Simpson’s rule and patients were stratified into four groups: LVEF < 30%—severe impairment of systolic function, LVEF between 30 and 39%—moderate systolic dysfunction, LVEF between 40 and 55%—mild systolic dysfunction, and LVEF > 55%—normal systolic function.

Systolic pulmonary artery pressure was calculated according to standardized criteria. In relation to measured values, patients were stratified into normal pulmonary artery pressure (PAPs < 25 mmHg), high normal pulmonary pressure (PAPs 25–29 mmHg), moderate pulmonary hypertension (PAPs 30–40 mmHg), and severe pulmonary hypertension (PAPs > 40 mmHg).

The severity of valve regurgitation (aortic and mitral) was assessed by a semiquantitative method and stratified as mild, moderate, or severe. Patients with aortic stenosis were divided into 3 groups according to peak gradient: mild aortic stenosis (gradient between 30 and 49 mmHg), moderate aortic stenosis (gradient between 50 and 70 mmHg) and severe aortic stenosis (gradient > 70 mmHg). Right ventricular function evaluated by Tricuspid Annular Plane Systolic Excursion (TAPSE) was defined as normal (TAPSE > 20 mm), mild dysfunction (TAPSE between 20 and 15 mm) and severe dysfunction (TAPSE < 15 mm).

### 2.2. Statistical Analysis

The parameters considered were expressed as mean values and standard deviations. We had no missing data. Categorical variables were expressed as distribution frequencies. In the comparison between groups, continuous parameters were compared by Student’s Test for unpaired data while the χ^2^ or Fisher’s Exact Test was used for categorical variables. The influence of the different parameters on mortality was evaluated by multivariate logistic regression analysis. Pulmonary artery pressure and LVEF were entered as continuous values. In the logistic regression model, we entered preoperative covariates whose results were statistically significant during the univariate analysis. Odds ratios were adjusted for clinically relevant confounders. Statistical analysis was performed with the use of a statistical software program (SPSS 26, Inc., Chicago, IL, USA).

## 3. Results

This prospective observational study included 2272 patients admitted to a tertiary teaching hospital between 1 January 2019 and 31 December 2024 with a diagnosis of a low-energy hip fracture. The mean age was 83.8 years. More than seventy percent were females (72.6%) with a female-to-male ratio of 2.65:1. The types of fracture are reported in Table 1. A significant functional impairment, expressed as loss of 2 or more BADL, was found in 28.2%. Twenty-one percent had cognitive impairment. More than half of the patients (62.4%) had at least two comorbidities. Time to surgery was less than 48 h from trauma in 76.5% of patients. Only 24 (about 1%) were treated conservatively. The characteristics of patients are reported in Table 1.

**Figure 1:**
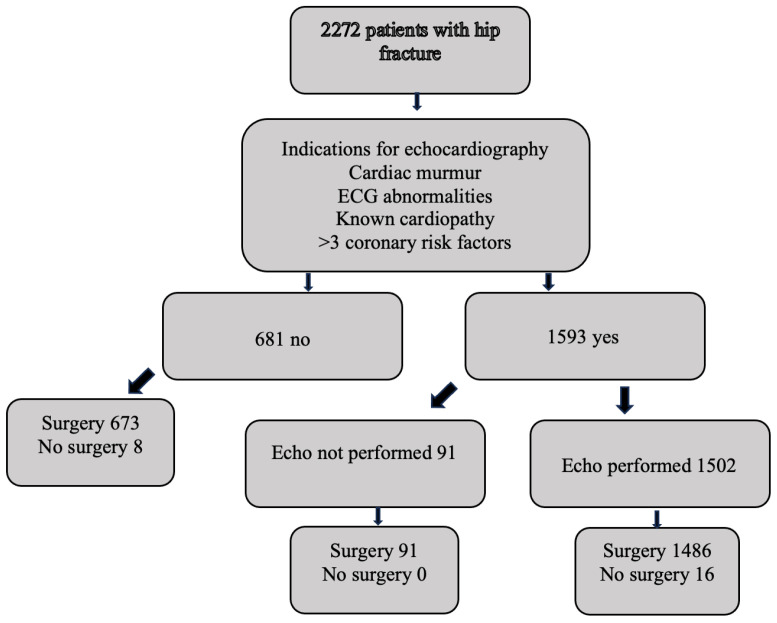
Flow chart of the study.

According to the study protocol, 1593 patients had indications to perform preoperative echocardiography. Echocardiography was performed before surgery in 1502 patients. Ninety-one, despite clinical indication, were not examined, mainly due to organizational problems. The other 681 without clinical indications, the “NO ECHO” group, underwent only routine ECG without further preoperative cardiological evaluation. A total of 86% of echocardiograms were performed within 24 h of trauma. In Figure 1 the flow chart of the study is reported.

Mean age was higher in the group with indication for echocardiography (85.4 ± 7.2 years in the ECHO group vs. 80.4 ± 11.2 in the NO ECHO group). Both groups had a higher female prevalence (71.3% in ECHO and 74.87% in NO ECHO respectively); however, the proportion was lower in those with indication for echocardiography who did not undergo examination. In patients not evaluated with a preoperative echocardiogram, time to surgery was on average 2.1 ± 2.3 days in comparison with 2.7 ± 4.1 days in those who underwent an echocardiogram. A similar time to surgery was found in patients with indication for examination in whom the exam was not performed.

Detailed data about the type of fracture and surgical treatment are reported in Table 2.

Functional impairment defined as preserved BADL < 4 was found in 22.8% of patients who did not undergo an echocardiogram and 31.09% of patients who underwent echocardiography. Two or more comorbidities were found in 72% of patients with indication for echocardiography in comparison to 42% of patients without. The frequencies of the different comorbidities found in the three groups are shown in Table 3.

**Figure 2:**
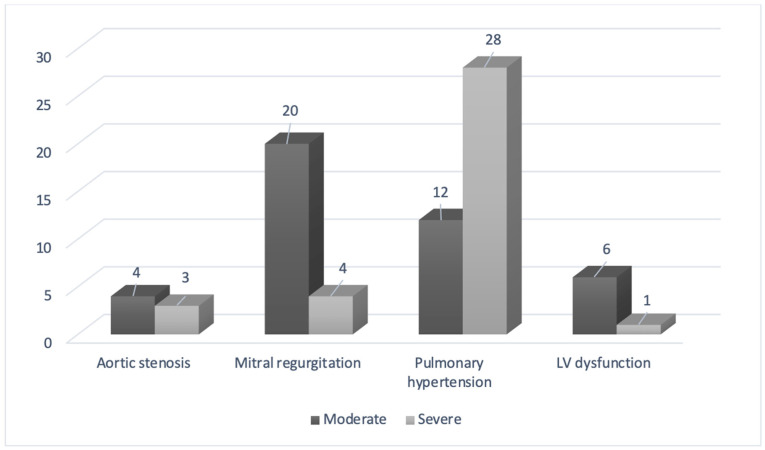
Frequency distribution of moderate–severe echocardiographic abnormalities.

In the echocardiographic group, left ventricular dysfunction was appreciable in 24.6%, while severely depressed ventricular function occurred in 19 patients. Moderate–severe aortic stenosis and mitral regurgitation were found respectively in 7 and 23%. Moderate to severe pulmonary hypertension was observed in 40.6%. Only 18 patients showed reduced RV function (TAPSE < 15). Twenty five percent had diastolic pattern abnormalities. The echocardiogram led to a modification to the anesthetic strategy (general vs. spinal anesthesia) in 13% of patients and to the need for ICU stay in the 24 h after surgery in 20%. Aortic stenosis accounted for 70% of cases, a marked reduction in left ventricular function for 27% and finally severe mitral valve disease for the remaining 3% (Figure 2).

In-hospital mortality was 2% in patients who did not undergo echocardiography, 7.3% in patients who underwent echocardiography and finally 10% in patients with indication for echocardiography in whom examination was not performed. In each group, patients discharged alive were younger and more frequently female. Time to surgery was longer in patients who died in hospital, and they also had a lower functional capacity (Table 4). Anemia and cognitive impairment were the only factors associated with mortality in patients who did not undergo echocardiographic examination (Table 5). In patients who underwent echocardiographic examination, anemia, atrial fibrillation, heart failure, COPD, cognitive impairment, >2 comorbidities and finally renal failure were significantly more frequent in patients who died in hospital in comparison to those discharged alive.

In Table 5 and Table 6, the different comorbidity rates in patients of the non-echo group and in patients with indication who either underwent echocardiography or did not, respectively, are reported.

**Figure 3:**
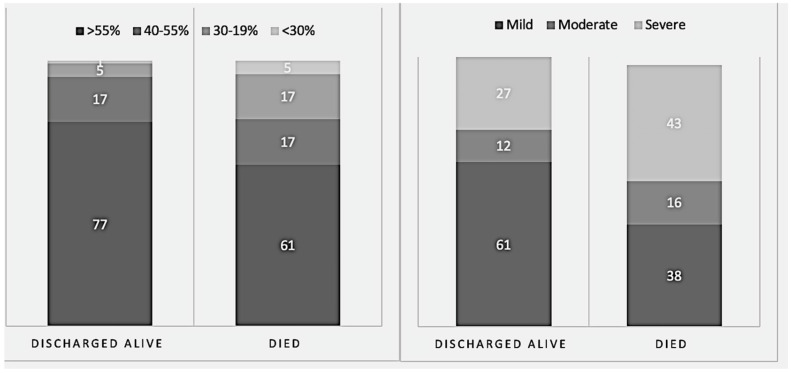
Distribution of LVEF and pulmonary artery pressure in patients discharged alive and who died in hospital. Numbers indicate the “Percent” distribution instead of distribution.

Considering echocardiographic parameters, moderate and severely depressed left ventricular function was found in 5.5% and 1.0% of patients discharged alive in comparison to 17.3% and 4.5%, respectively, of patients who died in hospital (*p* < 0.0001) (Figure 3). Moderate to severe mitral regurgitation was found in 22.8% of patients discharged alive and in 31.8% of patients who died (*p* = 0.0014). Thirty-nine percent of patients discharged alive had moderate to severe pulmonary hypertension in comparison to 59.1% of patients who died (*p* = 0.0018). We did not find any statistically significant difference in the rate of aortic stenosis and regurgitation, diastolic abnormalities or TAPSE between patients discharged alive and patients who died in hospital. In the multivariate analysis, age, time to surgery, anemia at admission, and cancer were independently related to in-hospital mortality while preserved functional capacity, expressed as maintained BADL, was related to survival. Among echocardiographic parameters, increased in-hospital mortality was associated with reduced EF (OR 1.45, 95%, CI 1.06–1.93, *p* = 0.02) and increased pulmonary pressure (OR 1.22, 95% CI 1.02–1.47, *p* = 0.03) (Table 7).

## 4. Discussion

AHA/ACC guidelines for cardiac evaluation in patients who need non-cardiac surgery suggest assessment of left ventricular function (class I, N-RB) in patients with new dyspnea, physical examination findings of HF, or suspected new/worsening ventricular dysfunction, as well in patients with aortic stenosis, to guide perioperative management [6]. Otherwise, ESC guidelines recommend echocardiography in patients with poor functional capacity and/or high NT-pro-BNP/BNP or murmurs detected before high-risk surgery (class I B) and in patients with suspected new CVD or unexplained signs or symptoms before high-risk NCs (class IIa, B) [5]. Patients with fragility hip fractures represent a major challenge in preoperative evaluation since significant cardiopathy may be masked by poor functional capacity related to age and frailty as well to cognitive impairment. Therefore, they may benefit most from preoperative echocardiography. Results of previous investigations on preoperative echocardiography in patients with hip fracture are contrasting. Since both survival and functional results are related to time to surgery, the best results are obtained when surgery is performed within 24–48 h of trauma. The main concern with preoperative echocardiography is related to an unacceptable delay in treatment while awaiting examination and the related increase in complications and mortality [12,13,14]. Canty et al. [15] compared patients at increased cardiac risk who underwent preoperative transthoracic echocardiography (TTE) to a randomized cohort who had similar cardiac risk but did not undergo preoperative TTE. In the TTE group, mortality was lower at both 30 days and 12 months postoperatively (respectively 4.7% vs. 15.2%, *p* = 0.047, and 17.1% vs. 33.3%, *p* = 0.031). In the study by Chen et al. [19], the mortality rate was 2.8% while the postoperative cardiac complication rate was 7.6%. History of coronary artery disease (CAD) and presence of aortic stenosis were independent predictors of postoperative cardiac complications. Moreover, the authors reported that age, a history of CAD, aortic stenosis and LVEF <50% were independently related to in-hospital mortality.

A retrospective study included 1453 patients (51% male; age, 67 ± 16) who underwent TTE before intermediate- or high-risk major abdominal surgery or orthopedic surgery [3]. The primary endpoint was major adverse events (MAEs), i.e., all-cause mortality and major adverse cardiovascular–cerebral events (MACCEs) at a follow-up of 56 days. Mortality was 2.4% and the MACCE rate 1.2%. Higher average E/e’ values, lower left ventricular (LV) ejection fraction, and higher prevalence of significant mitral regurgitation (MR), as well as moderate–advanced chronic kidney disease (CKD), were associated with MAEs. A recent case–control study matched 113 hip fracture patients who did or did not undergo preoperative TTE [20]. Indication for TTE was adequate in 71%. The TTE group had longer time to surgery and LOS, on average 20 h and 2 days respectively, in comparison to the non-TTE group (*p* < 0.0001). There was a higher 90-day mortality for the TTE group (odds ratio 4.4, 95% confidence interval 1.3–14.7, *p* < 0.03) but no survival difference at 2 years. In a large retrospective study from Japan, 34,679 (52.1%) of 66,620 hip fracture patients underwent preoperative echocardiography screening [16]. Propensity score matching created a matched cohort of 25,205 pairs of patients. There were no in-hospital mortality differences between the two groups (screened versus non-screened: 417 [1.65%] vs. 439 [1.74%]). Preoperative echocardiography was not associated with reduced postoperative complications and intensive care unit admissions. In our study, echocardiography was performed according to protocol in patients with newly detected or known systolic murmurs, ECG abnormalities, known heart disease or the presence of ≥3 coronary risk factors. Ninety-four percent of those with clinical indications underwent examination. We did not find a difference in time to surgery between patients who did or did not undergo echocardiography, since our organization allowed bed-side examination to be performed at the time of admission to the multidisciplinary ward. In more than 20% of those who underwent echocardiography, the perioperative strategy was changed according to ultrasonographic findings: in 13%, the anesthesiologic strategy was modified from spinal to general anesthesia; in the remaining patients, postoperative ICU was required. These data suggest the usefulness of echocardiography in perioperative planning; however, the lack of randomization does not allow us to assess whether the clinical outcomes may be modified by the echo-induced change in perioperative strategy.

TTE patients had a higher mortality, as expected, since inclusion criteria identified them as a high-risk surgical group. Mortality in patients with indication for but who did not undergo echocardiography was 10% in comparison to 7.3% in those who underwent examination. The small number of patients in the group who did not have an echocardiogram and the absence of randomization do not allow us to draw conclusions.

In the multivariate analysis, in-hospital mortality was associated with reduced LVEF (OR 1.45, 95% CI 1.06–1.93, *p* = 0.02) and increased pulmonary pressure (OR 1.22, 95% CI 1.02–1.47, *p* = 0.03). The association of depressed left ventricular function with increased in-hospital mortality has been previously described [3,20] and our data confirm these findings. Less is known about the relation between increased pulmonary artery pressure and mortality. The few available pieces of data report a four-fold increase in risk of mortality after non-cardiac surgery in pulmonary hypertension of any etiology in comparison to patients without PH [21]. In our study we found moderate–high pulmonary artery pressure in about 40% of patients. Several factors may contribute to this figure. First, a relation between aging and an increase in pulmonary artery pressure has been reported by Lam et al. [22]. Increased right ventricular afterload despite normal left ventricular function was associated with worse clinical outcomes [23,24]. Second, a significant number of our patients had preserved left ventricular function heart failure, in particular associated with atrial fibrillation. Third, moderate to severe aortic and mitral valve diseases were detected in about 30%. Therefore, it is not surprising to find elevated pulmonary artery pressure in a substantial proportion of the studied population. By evaluating the association between pulmonary hypertension and mortality, we must underline that a single value was taken before surgery, and hemodynamic variability related to volume changes due to bleeding and/or fluid administration, as well as adrenergic activation, may have influenced calculation. Although these data need to be further confirmed, we suggest careful preoperative treatment with the aim to decrease the risk of pulmonary congestion in these patients.

Other factors related to in-hospital mortality were age, time to surgery, anemia at admission, functional impairment, and cancer in agreement with the literature [8,10,11].

What is known about preoperative echocardiography in patients who need hip fracture surgery is the result of observational studies with different protocols and inclusion criteria, which therefore do not give definitive indications. An ongoing multicenter trial plans to enroll 2000 adults with hip fractures [25]. Participants will be randomized before surgery to receive either focused cardiac ultrasound (FCU) as part of their preoperative assessment or routine care without FCU with the aim of determining whether preoperative FCU of hip fracture patients reduces serious postoperative complications, improves the quality of recovery, improves quality of life, and is cost-effective.

### Limitations

The main limitation of this study was its single-center observational design. The indications for echocardiography included in the protocol were associated with a selection bias which created groups with clearly different risk profiles. Nevertheless, it must be emphasized that the main aim of the study was to evaluate whether in a multidisciplinary hip fracture unit, echocardiography delays the time to surgery. This objective was reached. As a secondary endpoint we tried to evaluate the association between echocardiographic abnormalities and in-hospital mortality. Depressed left ventricular ejection fraction and calculated systolic artery pressure were associated with higher mortality. Several factors may affect a single echo preoperative estimation of systolic pulmonary pressure; nevertheless, the few pieces of data in the literature agree with our findings. Regarding the mild difference in mortality rate found between patients with indication for echocardiography who did or did not undergo examination, the absence of randomization, the small number of events in patients who did or did not undergo echo, and finally the significant longer time to surgery found in this group, related to worse clinical conditions needing stabilization (7/9 had severe heart failure), do not allow for any causal consideration. They may be a matter for further investigations.

## 5. Conclusions

Results from our study confirm that in a large-volume, well-organized multidisciplinary hip fracture unit, the execution of echocardiography does not affect time to surgery. Moreover, we demonstrated that impaired left ventricular ejection fraction and pulmonary hypertension are associated with increased mortality. At present several pieces of evidence exist that echocardiographic findings have direct implications for perioperative management and risk stratification. Further investigations are needed to evaluate whether these changes may affect clinical outcomes.

## Figures and Tables

**Table 1:** Clinical characteristics of enrolled patients.

Total	2274	COMORBIDITIES	
Gender (N°, % female)	1637 (71%)	Atrial fibrillation	526 (23.1%)
Age (mean)	83.8 ± 8.7	COPD	208 (9.1%)
TYPE OF FRACTURE		Diabetes	405 (17.8%)
Neck femur	1106 (48%)	Cancer	467 (20.5%)
Pertrochanteric	1033 (46%)	Heart failure	184 (8.1%)
Subtrochanteric	135 (6%)	Dementia	476 (20.9%)
TYPE OF INTERVENTION		CAD	361 (15.9%)
Intramedullary nail	1113 (49%)	Peripheral arteriopathy	545 (23.9%)
Cephalic prosthesis	568 (24%)	Hypertension	1464 (64.3%)
Total prosthesis	338 (15%)	Renal failure (creatinine clearance < 25 mL/min/1.73 m^2^)	305 (13.4%)
Screws	228 (11%)	Transfusion	1125 (49.4%)
No surgery	24 (1%)	Anemia	152 (6.7%)
TIME TO SURGERY		Comorbidities ≥ 2	1419 (62.4%)
<48 h	1649 (76.5%)	PRESERVED BADL < 4	575 (28.2%)

**Table 2:** Clinical characteristics of the three groups.

	No Echo = 681	No Echo with Indication = 91	Echo = 1502	*p*
M	170 (25%)	38 (40%)	431 (28.7%)	0.02
F	511 (75%)	53 (60%)	1071 (71.3%)	
Mean age (years)	80.4 ± 11.2	84 + 8	85.4 ± 7.9	<0.0001
Type of fracture Neck Pertrochanteric Subtrochanteric	360 (52.7%) 291 (43.3%) 30 (4.1%)	44 44 3	702(46.8%) 695 (46.4%) 102 (6.8%)	=0.01
TIME TO SURGERY (days)	2.1 ± 2.3	2.9 + 3.7	2.7 ± 4.1	<0.001
SURGERY < 48 H (%)	80%	75%	74%	<0.0001
PRESERVED BADL < 4	171 (22.8%)	14 (15%)	411 (31.1%)	<0.001
Anemia < 10 g/dHb	113 (14%)	8 (9%)	31 (4.1%)	ns
Type of surgery No surgery	8 (1%)	0 (0%)	16 (1%)	0.05
Intramedullary nail	320 (46%)	50 (54%)	743 (49%)	
Cephalic prosthesis	132 (19%)	17 (19%)	422 (28%)	
Total prosthesis	130 (19%)	18 (20%)	190 (13%)	
Screws	91 (15%)	6 (7%)	131 (9%)	

**Table 3:** Distribution of comorbidities in the three groups.

COMORBIDITY	NO ECHO n 681	NO ECHO with Indications n 91	ECHO n 1502	*p*
ATRIAL FIBRILLATION	0	57 (62%)	469 (31%)	<0.0001
COPD	25 (4%)	11 (12%)	167 (11%)	<0.0001
DIABETES	99 (14%)	17 (18%)	299 (20%)	ns
CANCER	113 (16%)	27 (29%)	327 (21%)	ns
HEART FAILURE	0	27 (29%)	169 (11%)	<0.0001
DEMENTIA	141 (20%)	21(23%)	320 (21%)	ns
CORONARY DISEASE	0	28 (30%)	337 (22%)	<0.0001
PERIPHERAL ARTERY DIS.	103 (15%)	30 (33%)	412 (27%)	<0.0001
HYPERTENSION	358 (52%)	59 (64%)	1045 (69%)	<0.0001
≥2 COMORBITIES	263 (38%)	70 (73%)	1088 (72%)	<0.0001
RENAL FAILURE	43 (6%)	13 (15%)	263 (17%)	0.002
TRANSFUSION	284(41%)	42 (44%)	811 (53%)	<0.0001

**Table 4:** Comparison of clinical characteristics between patients who were discharged alive or died in hospital in the three groups (§ *p* = 0.004, * 0.0001, ° *p* = 0.005 (difference in the same group).

	NO ECHO (681)		NO ECHO with Indications (91)		ECHO (1502)		
	Discharged Alive (666–98%)	Died (15–2%)	Discharged Alive (82–90%)	Died (9–10%)	Discharged Alive (1392–92.7%)	Died (110–7.3%)	*p* <0.0001
Female	500 (75%)	9 (60%)	50 (60%)	5 (58%)	1006 (72%)	65 (59.1%)	<0.0001
Male	166 (25%)	6 (40%)	32(40%)	4 (42%)	386 (28%)	45 (40.9%) §	
Mean age	79.8 ± 11.4	86.1 ± 6.8 °	81.3 ± 7.5	87.2 ± 7 *	85.4 ± 7.9	88.9 ± 6.4 *	0.005
Time to surgery (gg)	2.1 ± 2.3	3.1 ± 6.8	2.5 ± 2.0	9.3 ± 13	2.6 ± 4.1	4.0 ± 10 §	<0.001
SURGERY < 24 h (%)	80%	61% *	64%	4.4%	74%	52% *	<0.0001
PRESERVED BADL < 4	94 (13%)	9 (60%) *	21 (25%)	5 (55%)	517 (37%)	51 (58%) *	<0.0001

**Table 5:** Distribution of comorbidities in patients who were discharged alive and died in hospital in the non-echo group.

Comorbidity	Discharged Alive (666)	Died (15)	*p*
Anemia	100 (15%)	9 (41%)	<0.0001
COPD	24 (4%)	3 (12%)	ns
Diabetes	105 (17%)	3 (12%)	ns
Cancer	139 (20%)	7 (29%)	ns
Dementia	137 (20%)	11 (45%)	0.002
Peripheral vascular dis	141 (21%)	7 (29%)	ns
Hypertension	353 (53%)	13 (54%)	ns
Comorbidities ≥ 2	256 (38%)	14 (58%)	ns
Renal failure	39 (6%)	3 (12%)	ns
Transfusion	277 (41%)	12(50%)	ns

**Table 6:** Distribution of comorbidities in patients who were discharged alive or died in hospital, with indication for echocardiography, and who underwent examination or did not. # *p* = 0.001, * *p* = 0.02, ° *p* = 0.001 (difference in the same group).

	ECHO (1502) and No ECHO with Indication (91)				
Comorbidity	Discharged Alive n 1392	Died n 110	Discharged Alive n 82	Died n 9	*p*
Atrial fibrillation	423 (30%)	46 (42%) *	55 (60%)	2 (22%) *	0.0001
Anemia	86 (6%)	24 (21%) °	4 (5%)	5 (55%) #	0.0001
COPD	146 (10%)	21 (19%) *	7 (9%)	2 (22%)	0.02
Diabetes	279 (20%)	20 (18%)	15 (18%)	1 (11%)	ns
Cancer	292 (21%)	35 (32%) *	21 (25%)	3 (33%)	0.04
Heart failure	147 (10%)	22 (20%) #	14 (17%)	7 (77%) #	0.0001
Dementia	286 (20%)	34 (30%) *	13 (16%)	4 (44%) *	0.02
Coronary artery disease	305 (22%)	32 (29%)	22 (26%)	3 (33%)	ns
Peripheral vascular dis	375 (7%)	37 (33%) *	18 (21%)	6(66%) #	0.002
Hypertension	977 (70%)	68 (62%) *	53 (64%)	6 (66%)	0.02
Comorbidities ≥ 2	992 (71%)	96 (87%) °	63 (76%)	7 (77%)	0.004
Renal failure	225 (16%)	38 (34%) °	10 (12%)	3 (33%)	0.0001
Transfusion	757 (54%)	54 (49%)	37 (45%)	5 (55%)	ns

**Table 7:** Multivariate analysis.

	Odds Ratio	95% CI	*p*
Age	1.07	(1.031 to 1.11)	0.0005
Time to surgery	1.03	(1.01 to 1.07)	0.0423
BADL > 4	0.85	(0.74 to 0.98)	0.0286
Anemia < 10 g/dL Hb	3.41	(1.82 to 6.37)	0.0001
Cancer	1.95	(1.18 to 3.21)	0.0084
Left ventricular ejection fraction	1.45	(1.07 to 1.99)	0.0189
Pulmonary hypertension	1.22	(1.02 to 1.47)	0.0257

## Data Availability

Data were collected from our digital records and are available on request.
